# Bone Quality: An Empty Term

**DOI:** 10.1371/journal.pmed.0040027

**Published:** 2007-03-06

**Authors:** Harri Sievänen, Pekka Kannus, Teppo L. N Järvinen

Although the concept of “bone quality” is at least 15 years old [1], the term has recently sparked much discussion and debate among clinicians and clinical researchers [2–5]. At a recent National Institutes of Health conference on bone quality, the term was defined as: “The sum total of *characteristics of the bone* that influence the *bone's resistance to fracture*” [6].

## Where Did the Definition Come From?

This definition arose from the results of multicenter clinical trials that evaluated the effects of two classes of drugs—antiresorptive bisphosphonate therapy (alendronate and risedronate) and selective estrogen receptor modulator therapy (raloxifene)—on the prevention of osteoporotic fragility fractures [7,8]. While these studies reported consistent reductions in the incidence of fractures, the treatment effects could not be explained by contemporary changes in dual X-ray absorptiometric bone mineral density (BMD), the present clinical standard of bone fragility. These conflicting findings led to speculation that the antiresorptive drugs had additional skeletal effects upon a feature of the bone called “bone quality” [7–12].

The idea of bone quality, and the explanation for the conflicting results, linked together two important notions: (1) antiresorptive drugs acted by suppressing bone turnover through inhibiting bone resorption, and (2) increased bone turnover (mainly the increased bone resorption, as detected by bone markers) compromises the bone strength through deteriorated bone microarchitecture (a trait that cannot be captured by BMD measurement but could potentially be improved by antiresorptive treatment) [4].

Bone quality is now a widely embraced concept that seems to offer a solution to the classic paradox of osteoporosis: while low BMD values are associated with increased relative risk of fracture at the *population level*, the predictive value of BMD in an *individual patient* remains quite marginal [13–15]. And to further support the concept of bone quality, inclusion of increased bone turnover in fracture-predicting models has somewhat improved the ability to predict fracture risk independently of BMD [8,16–19].

## Flaws in the Concept

Although the concept of bone quality might seem attractive for all of the reasons discussed above, nevertheless the notion has three major conceptual flaws.

### BMD and bone quality do not explain fractures.

First, although BMD indeed shows a strong correlation with whole bone strength in the laboratory setting (*r* up to ~0.9) [20], in the clinical setting, paradoxically, the overall proportion of various fragility fractures attributable to low BMD (indicating reduced bone strength) remains modest (from 0% to 44%) [15]. In other words, when looking at all types of fractures combined, over half occur among people who *cannot* be classified as having osteoporosis in the sense of the World Health Organization's operational definition of osteoporosis (BMD 2.5 standard deviations or more below the young adult reference level). In fact, BMD is only a modest risk factor for fractures; about 85% of the contribution to the fracture risk in general, or to the rise in fracture risk with age, is unrelated to BMD [15,21]. In other words, the concept of bone quality is invoked to explain fracture risk that cannot be attributed to BMD, but it seems impossible that bone quality could realistically explain 85% of all fractures.

### BMD and bone quality are largely inseparable.

Second, the concept of bone quality rests on a commonly held idea that BMD and bone quality would *independently* account for bone fragility in totality. But this idea is a fallacy. Basically, BMD reflects the bulk of material (bone mass) of which the bone, as an organ, is made [22]. BMD thus denotes a lumped measure of virtually everything within the measured bone site (i.e., bone cross-sectional size and dimensions, cortical thickness and porosity, trabecular thickness and number, mineralization of bone material), but it denotes nothing specifically. Thus, there is not much left to be accounted for by subtle architectural and material properties (i.e., factors that allegedly account for bone quality). This simply means that BMD and most bone quality characteristics, measurable in vivo, are intertwined and largely *inseparable*.

### Flaws in defining bone quality.

Third, the definition of bone quality is too imprecise, incorporating a pool of “non-BMD” indices of bone fragility (or, even more broadly, the portion of fracture risk that is not predicted by BMD [6]). Neither do we have an established measurement, indicator, or unit for bone quality. We don't even have criteria for defining “good” or “bad” bone quality.

## The Problem of Measurement

*“If you can not measure it, you can not improve it.”*

*Lord Kelvin*

In business and industry, quality is classically defined as “fitness for use” [23] or “conformance to requirements” [24]. Extrapolating from these standard business definitions of quality, “good” bone quality would mean a high level of resistance to fractures, or extrapolating even further [6], resistance to *all* factors accounting for fracture risk. One might then ask whether the likelihood of a fracture is solely dependent on bone strength, and accordingly, whether a fracture that resulted from mild or moderate trauma is a direct index of “bad” bone quality.

However, this kind of simplistic thinking ignores the fact that the etiology of any type of fracture among older adults is multifactorial, involving many *extra-skeletal* risk factors much stronger than the bone per se (measured by conventional BMD) [25–33]. In this respect, one should recall that bone quality, by definition, cannot be but a bone-based trait only [2].

Even if the problems with the definition of bone quality could be solved, the fundamental problem with the bone quality concept is common to all new diagnostic tests [34]: the clinical value of a new diagnostic test depends on whether it improves patient outcomes beyond the outcome achieved with current diagnostic tests (here, the BMD and other well-known risk factors of fractures). As mentioned previously, it is true that highly increased bone turnover has been shown to improve the ability to predict some types of fractures independently of BMD [8,17,18]. However, so far we have little proof that the biochemical markers of bone turnover would be able to make a clinically relevant impact on the predictive ability of fracture *independently* of the well-known risk factors of fractures.

For example, incorporating a single non-skeletal risk factor (gait speed) into the predictive equation along with BMD and bone markers was shown to clearly diminish the fracture predictive ability of bone markers [17]. Thus, it remains quite utopian to envision that a pure bone-derived measure (e.g., BMD complemented by bone quality) could ever cover all these extra-skeletal risk factors, too, and thus, predict solely the individual occurrence of fractures.

The prevailing understanding of bone quality supposes that the primary property of bones is their capacity to resist fractures. But this is a misconception. The human skeleton is basically a locomotive apparatus, which is continually adapting to habitual loadings [35,36] and is particularly fit for endurance activities [37]. Given the intrinsic locomotive function and the metabolic pressure to keep the skeleton light, there is a compromise between the bone's actual, functionally adequate strength and the maximum attainable strength. Bone has a great capacity to become substantially stronger through appropriate structural adaptations whenever needed to cope with increased functional demands [36]. However, while the skeleton can be reasonably well adapted to customary, functional loadings (Figure 1A), it is definitely not adapted to unusual loadings caused by occasional falls (or by other similar trauma-related events) [38]. Under such circumstances weak bone regions can become unduly stressed, possibly beyond their load-bearing capacity, initiating a fracture (Figure 1C). Fully in line with this fact, the relative risk of hip fracture can rise to up to 30 when the fall-induced impact directly hits the greater trochanter of the proximal femur [26,28,39]. It is thus quite understandable that the external loads from these non-habitual incidents cause most (up to ~90%) hip and wrist fractures [26,30,39] and also account for at least half of vertebral fractures [40–42].

**Figure 1:**
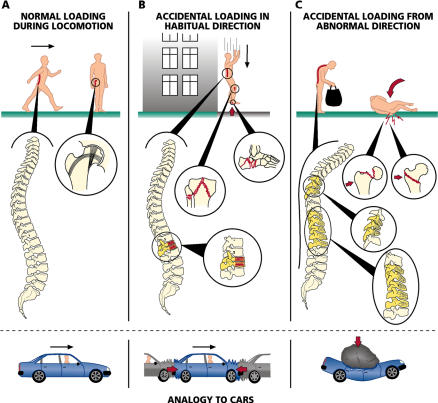
Bone Fractures, Car Accidents, and Direction of Impact Analogous to automobiles designed to run on their wheels, the human skeleton is adapted to bipedal gait and the resulting habitual locomotive loadings (Figure 1A). In terms of safety, the design of cars is optimized to keep the driver and passengers in the cockpit intact during collisions from the typical directions of impact, the front or rear (Figure 1B). However, a similar or even smaller force can cause profound damage to the cockpit if it comes from an atypical (unforeseen) direction (Figure 1C). Analogously, the capacity of the skeleton to resist fractures during accidents is generally good when the loading caused by a traumatic incident is a moderate magnification of the loading experienced during habitual activities (i.e., within the inherent safety margin of bone), except in some cases where the incident force exceeds the bones' capacity to withstand the loading without structural failure (Figure 1B). In many cases of older adults' fractures, however, the incident loading in terms of direction, rate, and magnitude is essentially different from the loading that bones are adapted to (Figure 1C). Such cases can be caused, for example, by careless lifting of a shopping bag with straight knees [42,48] or a sideways fall directly onto the hip [26,30].

## Conclusion

In the end, the only reasonable mechanism by which any bone-targeted medication reduces fractures is through increasing the whole bone strength (one way or another). Accordingly, if we were able to accurately determine whole bone strength of individuals on antiresorptive therapy, the alleged discrepancy underlying the concept of bone quality would not exist. As the whole bone strength provides the ultimate measure of true bone quality, the paradox of osteoporosis appears to simply stem from our inherent inability to determine directly the actual bone strength of an individual in vivo. However, this inability cannot be taken as a justification to introduce an obscure and ill-defined concept such as bone quality.

If it really must be used, the term bone quality should refer only to the capacity of bones to withstand a wide range of loading without breaking—though we already have a proper term for such capacity, the whole bone strength. Therefore, we must strive to reliably estimate the whole bone strength in vivo. In this context, the new 3-dimensional imaging techniques of the actual bone structure and macroanatomy seem an interesting and promising option [43–47] that will hopefully help in solving the important clinical issue of bone fragility in the near future.

## Abbreviations

BMD: bone mineral density
